# Enhanced Healing Activity of Manuka Honey and Nitrofurazone Composite in Full-Thickness Burn Wounds in the Rabbit Model

**DOI:** 10.3389/fvets.2022.875629

**Published:** 2022-05-23

**Authors:** Muhammad Fakhar-e-Alam Kulyar, Khurram Ashfaq, Amjad Islam Aqib, Kun Duan, Muhammad Asif, Zeeshan Ahmad Bhutta, Muhammad Shoaib, Samina Shabbir, Shah Nawaz, Muhammad Aamir Naseer, Iqra Sarwar, Muhammad Akhtar, Ayesha Safdar Chaudhry, Riaz Hussain, Hafiz Iftikhar Hussain, Yi Wu, Kun Li

**Affiliations:** ^1^Institute of Traditional Chinese Veterinary Medicine, College of Veterinary Medicine, Nanjing Agricultural University, Nanjing, China; ^2^College of Veterinary Medicine, Huazhong Agricultural University, Wuhan, China; ^3^Department of Clinical Medicine and Surgery, University of Agriculture, Faisalabad, Pakistan; ^4^Department of Medicine, Cholistan University of Veterinary and Animal Sciences, Bahawalpur, Pakistan; ^5^China Tobacco Henan Industrial Co. Ltd., Zhengzhou, China; ^6^Department of Surgery, University of Veterinary and Animal Sciences, Lahore, Pakistan; ^7^Laboratory of Biochemistry and Immunology, College of Veterinary Medicine, Chungbuk National University, Cheongju, South Korea; ^8^Key Laboratory of New Animal Drug Project, Gansu Province, Key Laboratory of Veterinary Pharmaceutical Development, Ministry of Agriculture, Lanzhou Institute of Husbandry and Pharmaceutical Sciences of Chinese Academy of Agricultural Sciences, Lanzhou, China; ^9^Key Laboratory of Development and Application of Rural Renewable Energy, Ministry of Agriculture and Rural Affairs, Biogas Institute of Ministry of Agriculture and Rural Affairs, Chengdu, China; ^10^Graduate School of Chinese Academy of Agricultural Sciences, Beijing, China; ^11^Department of Pathology, University of Agriculture, Faisalabad, Pakistan; ^12^Department of Pathology, Faculty of Veterinary Sciences, The Islamia University of Bahawalpur, Bahawalpur, Pakistan; ^13^Department of Pathology, Cholistan University of Veterinary and Animal Sciences, Bahawalpur, Pakistan

**Keywords:** Manuka, honey, Nitrofurazone, burns, natural remedies

## Abstract

Burns cause many significant changes in metabolism and inflammatory reactions, leading to poor regeneration in animals and humans. A list of medicines to treat burns is available in the market. But due to the high cost of these medicines, these are unaffordable, especially for farmers of middle-class families of Africa and Asia. Therefore, a low-cost complementary treatment has always been a topic of many researchers, and there is a dire need of time for the welfare of animals to save them. The current study was planned to scrutinize the therapeutic effects of Manuka honey and Nitrofurazone ointments on full-thickness burn wounds in the rabbit model. The healing efficacy was performed through wound contraction rate, hematological analysis, the thickness of dermis and epidermis, and collagen content percentage. Histopathology was performed after taking biopsy samples at the end of the research. Based on statistical analysis using wound healing time (days, D), the combination (MO + NT) resulted in a shorter period (27 D ± 1) than the average healing time of controlled (36 ± 2), Manuka ointment (31.33 D ± 1.52), and Nitrofurazone ointment (32 ± 1). A significant decrease in the count of red blood cell (RBC), mean corpuscular volume (MCV), and mean corpuscular hemoglobin (MCH) in all treatments was noticed mainly in MO + NT. Furthermore, burns induced a significant difference (*p* < 0.05) in the white blood cells (WBCs) count levels in the MO-treated group. While the level of platelets (PLTs) was not significantly different from the healthy control group. Histopathological assessment (epithelialization, fibrosis, and angiogenesis) of skin showed burn healing to be better in MO and MO + NT groups. In conclusion, the composite of Manuka honey with Nitrofurazone led to the faster recovery than other treatments.

## Introduction

Skin is a coated barrier that protects internal organs from harsh environments. This barrier is essential for maintaining the homeostasis of body fluids, controlling temperature, and protecting the body from infection. The skin also has immunological, nervous, and metabolic functions (1, 2), devastating issues that affect such a barring ability (3). According to a study, wounds account for 30% of the total emergency caseload in veterinary healthcare centers (4). Animals (especially pets) with severe burns need immediate access to professional facilities. Such modern resuscitation and emergency burn therapies are designed to mitigate systemic changes caused by the sudden destruction of skin barriers, but unfortunately, these facilities are not available in the developing countries of Asia and Africa. The majority of burns is unintentional that occurred at veterinary clinics, relating to the use of adjunct heat to reduce the risk of developing hypothermia. Veterinarians often encounter burns that are <20% of the animal's body surface. Thus, severe metabolic disturbances, such as electrolyte levels imbalances, red blood cells destruction, and high susceptibility toward systemic infection, might not be seen (5). But the use of such animals as a model is still a viable option in overall healthcare in translational medical studies (6). A list of medicines to treat burns, i.e., Nitrofurazone and silver sulfadiazine, is available in the markets. But due to the high cost of these medicines, most people cannot afford them. Hence, more traditional herbal products have been used in African and Asian middle-class families (7). According to the WHO statistics, 265,000 deaths annually occur due to burning, most of which happen in these regions (8). The herbal products used by these people seem moderately effective, less toxic, and less expensive than other market products. Honey has been used in folk medicine for a long time, but it has only recently been discovered by medical researchers that it can be used to treat both acute and long-term wounds.

Traditionally, honey has been used to treat burns (9) due to its antimicrobial properties, especially to cover up burn wounds. It has been shown to inhibit a wide spectrum of bacteria and is as efficient against antibacterial drugs resistant bacteria (10–12). Interestingly, one of the earliest experiments of honey's efficiency against antibiotic-resistant was conducted, using Manuka honey to treat a hydroxyurea-induced leg ulcer colonized by methicillin-resistant Staphylococcus aureus (MRSA). This research established that honey has antibacterial capabilities against resistant bacteria and may also aid in the healing process of wounds (13). Such antibacterial action of honey is due to its acidity, hydrogen peroxide contents, osmotic effects, nutritional and antioxidant contents, stimulation of immunity, and unidentified compounds (14). Generally, honey has glucose, maltose, sucrose, fructose, water, and a few other things, such as amino acids, organic acids, proteins, vitamins, acetylcholine, and flavonoids (15). Many scientific studies have been urged on its medicinal properties in Ayurvedic, Chinese and Roman traditions (14). Manuka honey is the purest form of unifloral honey that is derived from Manuka trees and Leptospermum scoparium, belongs to the family Myrtaceae in New Zealand and the Eastern region of Australia (16). It is dark honey as compared to other honey types. It contains the highest concentration of phenolic and flavonoid compounds (pinobanksin, pinocembrin, and chrysin) that have been identified as having potent reactive oxygen species (ROS) scavenging activity (17). Flavonoids polyphenolic compound is a group of secondary metabolites that naturally occur in the plant kingdom that possesses numerous pharmacological activities (18).

Nitrofurazone (C6H6N4O4) is a commercially available product used as an antimicrobial agent on different wounds, especially burn wounds (19). It has been reported to show bactericidal activity against many gram-positive and gram-negative bacteria, inhibiting bacterial enzymes involved in carbohydrate metabolism. It exhibits bactericidal activity against most pathogens that commonly cause surface skin infections (20). But Nitrofurazone (NT) therapy has a limit on its low aqueous solubility. The poor solubility of NT urges a slow-release rate, causing less penetration of an effective drug concentration into the skin. Moreover, due to its high permeability through the skin, NT remains for a limited time at the applied area (21). Others include sensitivity, itching, contact dermatitis, and longer time period (8).

The current experimental study explored the effects of topical application of Manuka honey ointment on full-thickness burns, in comparison with commercially available Nitrofurazone at the thoracoabdominal regions of rabbits. The evaluation parameters, such as healing time, contraction rate, hematological, and histopathological assessment, were compared with untreated thermally injured rabbits.

## Materials and Methods

### Treatment Preparations

The Manuka honey (batch no. GMH4765; lot no. KMH15816) was purchased from an online store, while Nitrofurazone was purchased from a local market. According to our one pilot project, all treatments were prepared according to their minimum effective levels. 1% Manuka honey ointment (MO), 1% Nitrofurazone ointment (NT), and 1% of their combination (MO + NT) were prepared by mixing them with 99 g of petroleum jelly. The quantity of both treatments was calculated on the base of their total percentage and purity. The final form of concerning ointments was then stored in the plastic bottles.

### Induction of Burn Wound

The ethics committee of the University of Agriculture Faisalabad, Pakistan approved this work (DGS/39257-60), in accordance with institutional and national guidelines. The nine rabbits from three different groups (MO, NT, MO + NT) were anesthetized by injectable anesthesia with xylazine (4 mg/kg) and ketamine hydrochloride at a dose rate of 35 mg/kg intramuscular (22). The thoracoabdominal side of the skin was shaved, and burn wounds were created using a metal plate (with a 10-mm diameter) heated at 100°C on an open flame of a Bunsen burner with equal pressure over the shaved area (23). After creating wounds, Ketoprofen (1 mg/kg) was injected as an analgesic to the rabbits (24). The administration of analgesic was performed for the next 3 days in order to attain an equal immune response for each individual.

### Experimental Design

**Figure 1:**
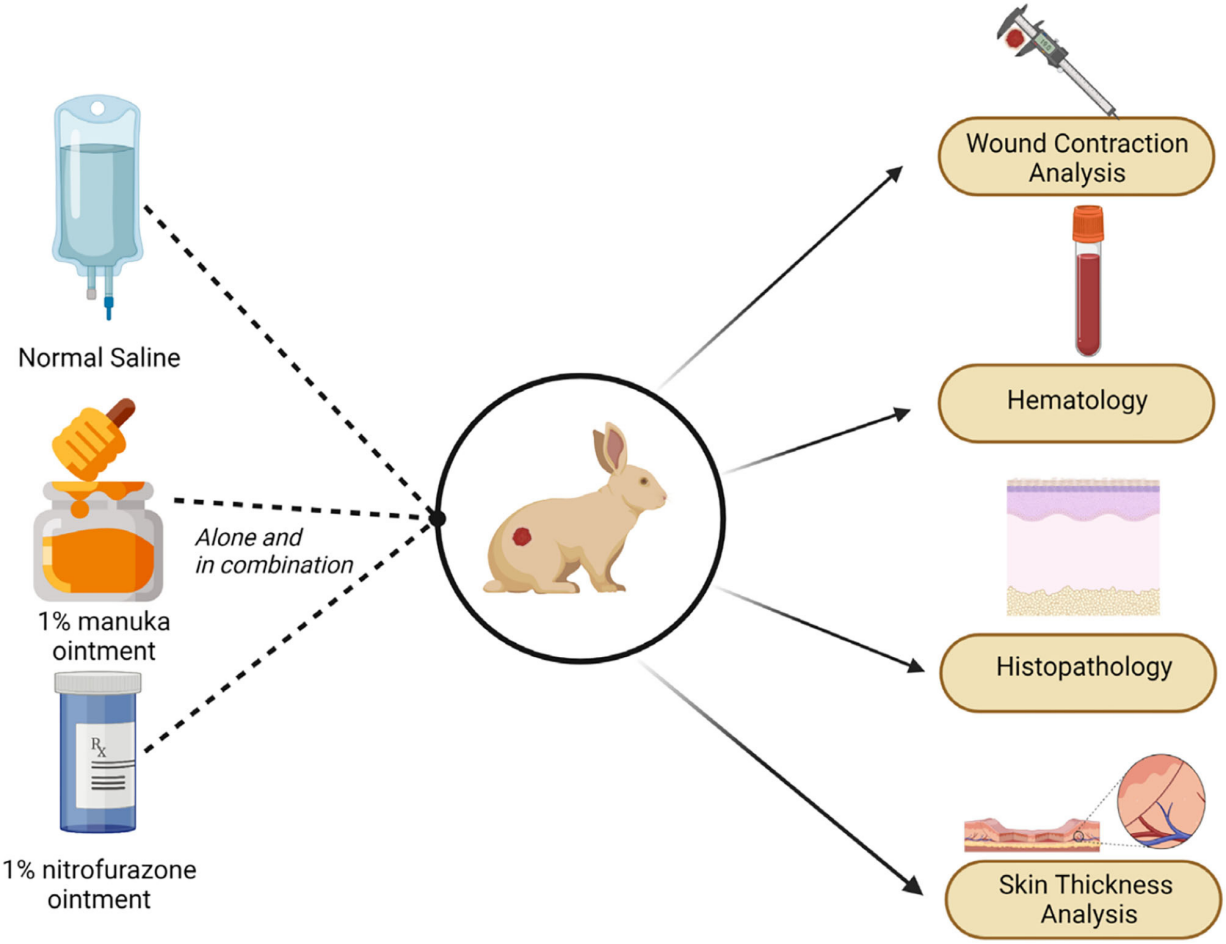
The schematic presentation of the experiment.

Fifteen male healthy rabbits (8–10 weeks old) weighing 1,500 g ± 20 g were randomly allocated to five treatment groups. Each treatment group was comprised of three rabbits. The acclimatization period of 3 days was provided to rabbits with the same conditions of environment and feeding in order to attain equal immune response for each individual. Rabbits were divided as follows: NS (negative control) received no treatment, PS (positive control) was allocated to normal saline, whereas MO (Manuka honey ointment), NT (Nitrofurazone ointment), and MO + NT were treated topically twice a day (Figure 1). The healing efficacy was performed through healing time, wound contraction rate, and hematological analysis on days 0, 7, 14, 21, and 28. Histopathology was performed after taking biopsy samples at the end of the research. All rabbits were fed pelleted food with *ad libitum* water throughout the experiment and were housed in the animal experimentation unit.

**Figure 2:**
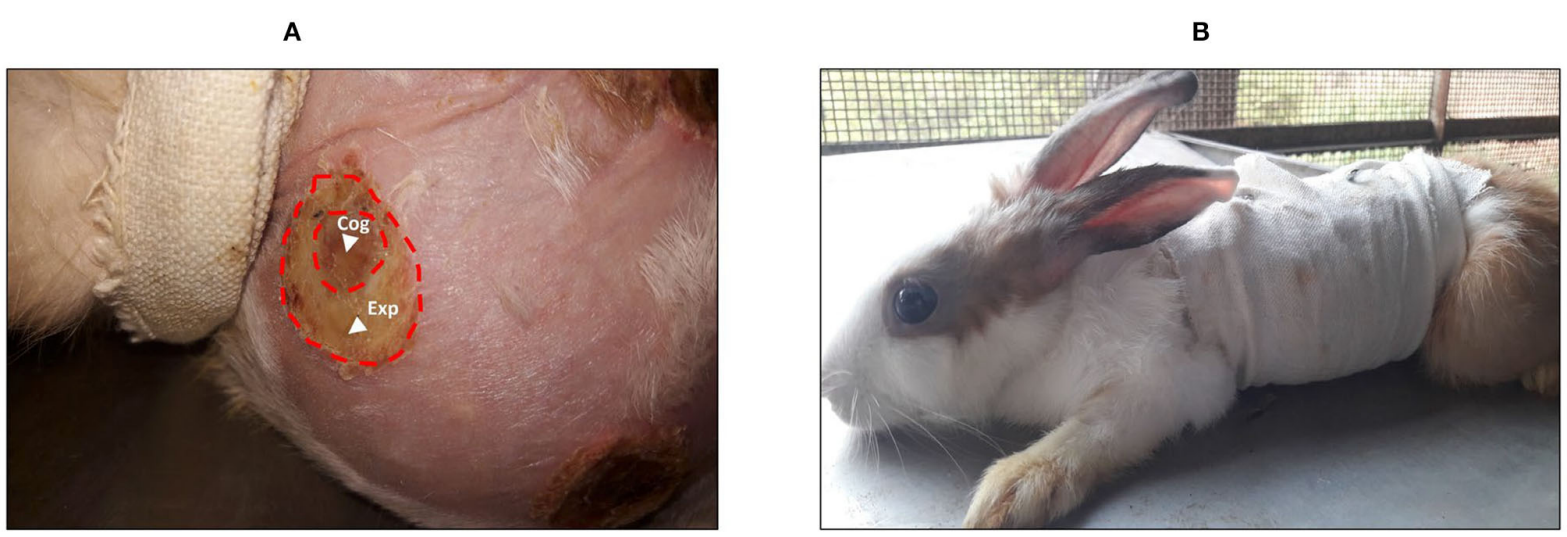
**(A)** Burn wound with the area of expansion that reveals a white color when compared with the coagulation zone. **(B)** A rabbit in a bandage after treatment protocol. Cog, coagulation zone; Exp, expansion zone.

In the preliminary test, the wound stopped increasing on the fourth day. Therefore, the observation period was set from days 0 to 4 after the injury (Figure 2A). After topical application of treatments, the wound was carefully covered with a bandage (Figure 2B).

### Measurement of Parameters

#### Healing Time

Healing time was considered from the time of wound induction to re-epithelialization. All the observations were estimated randomly (7, 14, 21, and 28) until the scar fell off (25).

#### Wound Contraction Rate

The wound healing was examined by digital photography every seventh day under general anesthesia (26). While the contraction rate percentage was determined by the following formula (27).


$$Contraction\;\%\;=\frac{\begin{array}{l} {}[Area\;on\;day\;zero\;\left(mm\right)\\ -Area\;on\;day\;measurement\;\left(mm\right)] \end{array}}{Area\;on\;day\;zero\;\left(mm\right)}\times 100$$

#### Hematological Analysis

Hematological analyses were accessed (0, 7, 14, 21, and 28) to compare the relative success rate of treatment groups (28).

#### Histopathological Analysis

Histopathological analyses were performed to compare the relative healing rate for different treatments. The parameters (thickness of the epidermis and collagen content) were evaluated during the histopathological examination, in which a tissue sample was collected at the wound site (at the end of the research). The different percentages were measured to indicate the degree of compression and orientation of the collagen fibers. Cells that are part of the immune system were also examined in the tissue that had been treated.

#### Statistical Analysis of Data

All statistical data analyses were carried out using ANOVA through SPSS statistical computer software (version 25.00). The recovery rates observed for various groups were compared using the independent sample *t*-test. The statistical significance level was set < 0.05.

## Results

### Healing Time

According to the *t*-test analysis, it is inappropriate to compare the values of two or more groups. Therefore, we performed ANOVA and *post-hoc* tests among four groups. The range of healing of full burns in the combination group was significantly different, and the frequency of treatment was also significantly improved. Rabbits were better in the first 2 weeks after that healing was gradually becoming better among different groups e.g., Manuka honey, Nitrofurazone, and the control group. The MO + NT resulted in a shorter period (27 D ± 1) than the average healing time of controlled (36 ± 2), MO (31.33 D ± 1.52), and NT (32 ± 1) (**Figure 4A**). Rabbits treated with the combination had significantly more prophylactic effects than untreated rabbits. Significant healing ornamentation (*p* < 0.05) within 14 days was noticed in two treatment groups (MO + NT and MO). There was also a significant reduction (*p* < 0.05) of wound size on day 7 in treated animals as compared to control group.

### Wound Contraction Rate

**Figure 3:**
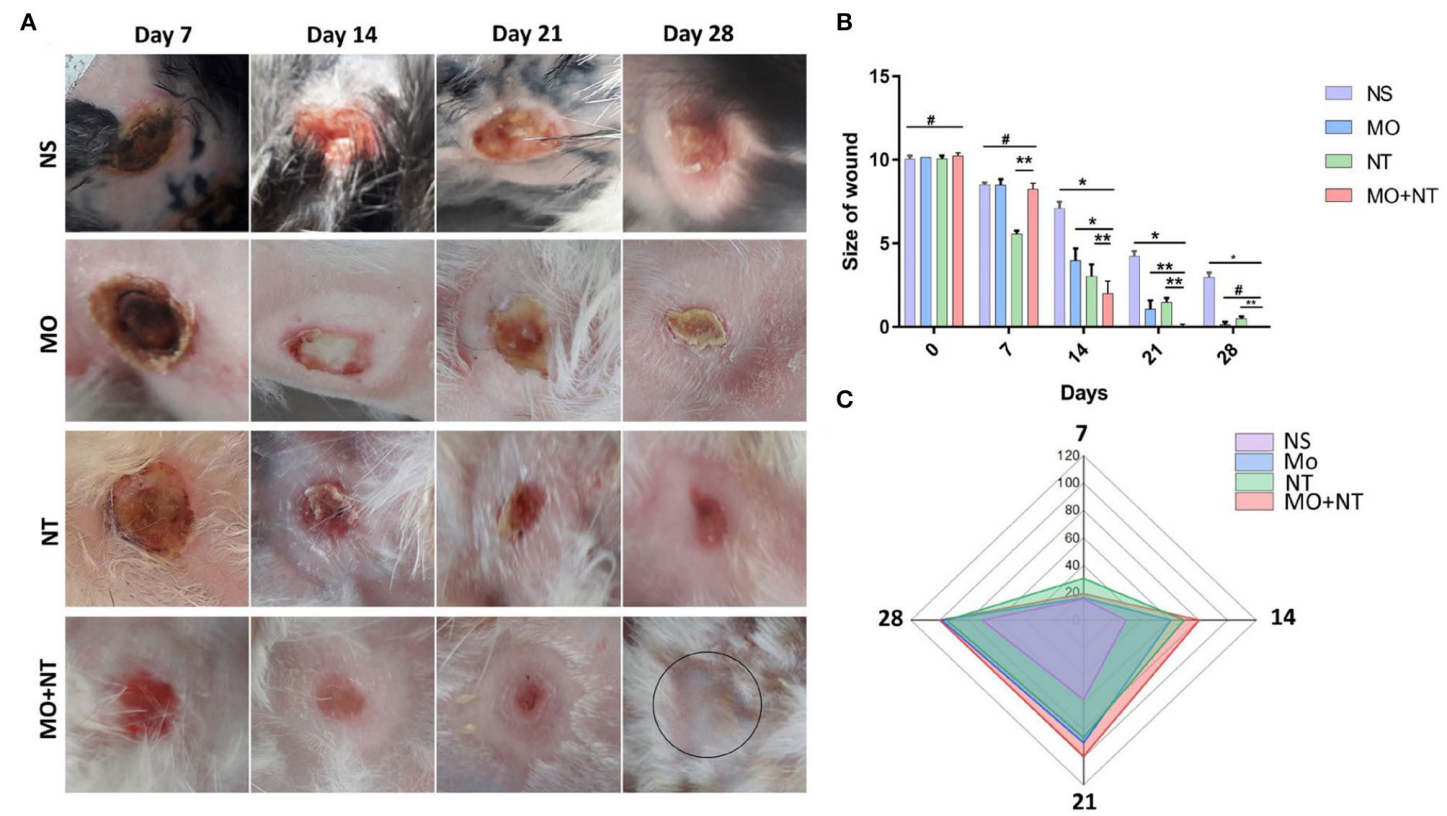
**(A)** Digital photographs of wounds taken on different days (7, 14, 21, and 28). NS, normal saline; NS, normal saline; MO, Manuka honey ointment; NT, Nitrofurazone ointment; MO + NT, Manuka honey + Nitrofurazone. **(B)** Size of wounds, **(C)** contraction rate percentage (*p < 0.05, **p < 0.01, ^#^p > 0.05).

The frequency of wound closure was higher in the groups that were treated with combination in rabbits. On day 14, rabbits of “MO + NT” group showed better granulation tissue formation. After 21 days, “MO + NT” showed remarkable healing and scarring (Figure 3A). The significance levels of “MO + NT” among other treatments, such as Manuka honey and Nitrofurazone, were 0.026 and 0.012, respectively. While the significant level with the control group was 0.00. Similarly, in the individual comparison of Manuka honey ointment with “MO + NT” and Nitrofurazone, the significance levels were 0.026 and 0.94, respectively (Figures 3B,C).

In the initial days, the wound area of all groups was increased. The enlarged area showed a bright white color as compared to the burned area. This area was white and became necrotic, corresponding to a stasis area. On the second day, the wound area of all groups continued to increase, and the new expansion area appeared white as compared to the coagulated site. On day 3, all groups formed a full red ring around the edges of the wound. This area corresponds to the hyperemic area, indicating increased blood flow. On the fourth day, all the wounds stopped expanding. All groups showed almost identical properties, but the MO + NT group showed intense redness at the wound's edges. The MO and the NT formed a scar at the edge of the wound.

### Hematological Analysis

**Figure 4:**
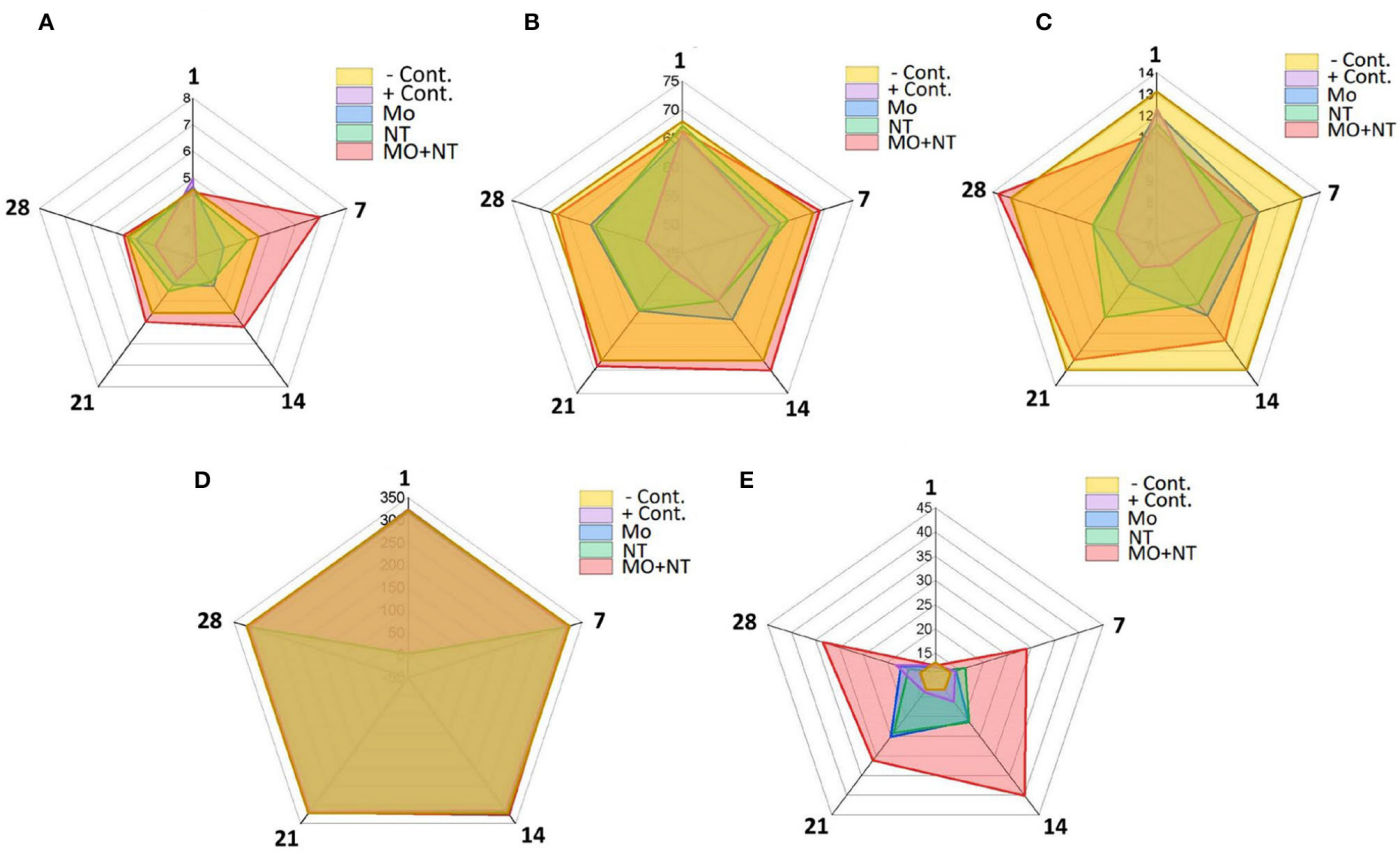
Hematological changes at different days (1, 7, 14, 21, and 28). **(A)** Ref blood cell (RBC) (10^12^/l), **(B)** MCV (Fl), **(C)** MCH (pg), **(D)** PLT (10^9^/l), and **(E)** white blood cell (WBC) (10^9^/l).

A significant decrease in RBC count, mean corpuscular volume (MCV), and mean corpuscular hemoglobin (MCH) on days 7, 14, 21, and 28 was noticed, except for MO + NT in all treatments. Furthermore, burns induced a significant difference (*p* < 0.05) in the WBCs count levels in the MO + NT treated group. The level of platelets (PLT) was not significantly different from the healthy control group (Figure 4). As it depends on heat damage and an increased level of lymphocytes. So, the number of neutrophils and eosinophils did not change as compared to the control group.

### Histopathological Analysis

**Figure 5:**
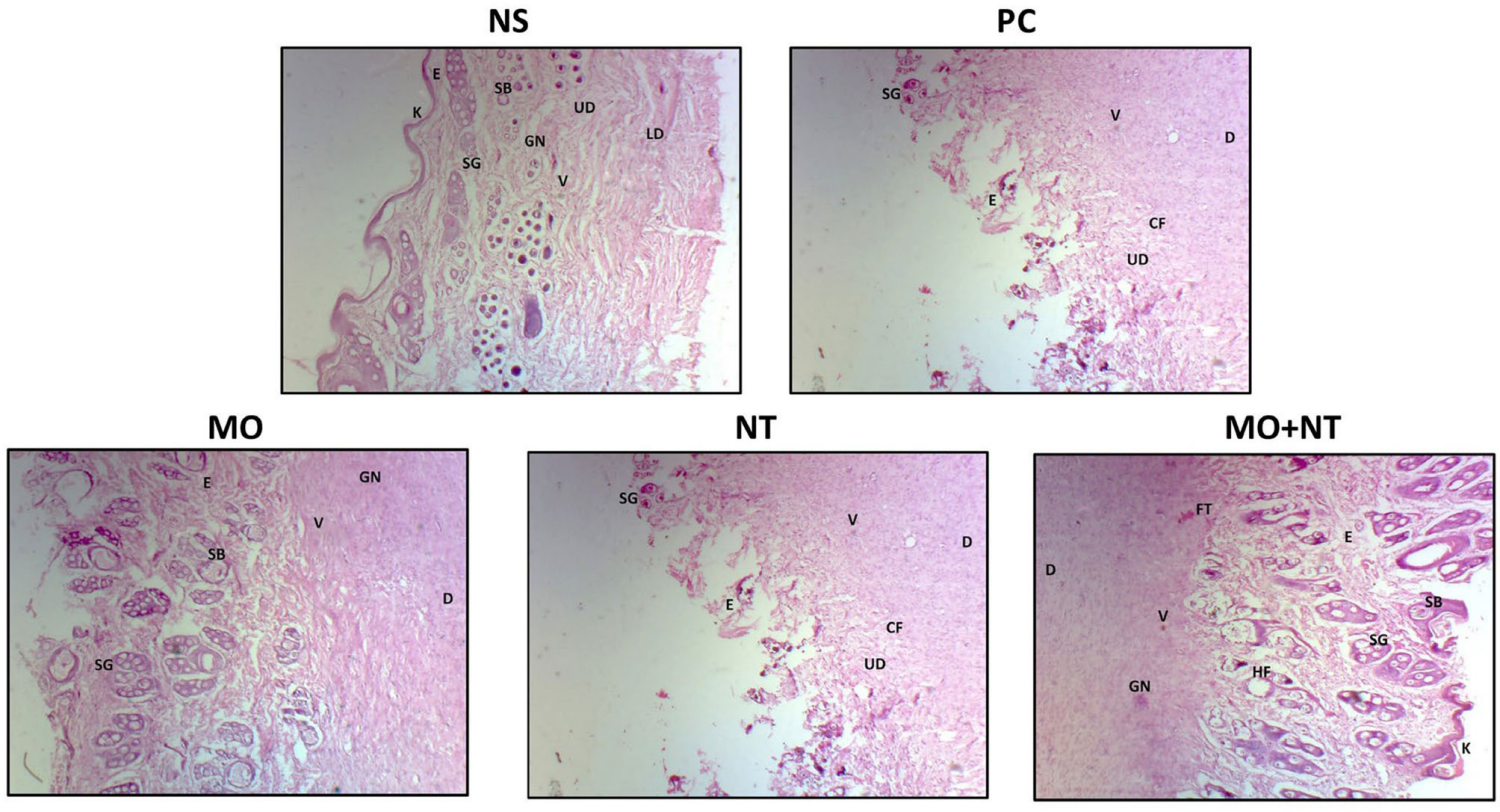
Histopathology of different treatments. E, epidermis; D, dermis; UD, upper dermis; LD, lower dermis; SG, sweat glands; SB, sebaceous glands; V, newly farmed blood vessels; GN, granular tissues or granulation; HF, hair follicle; FT, fibrous tissue or collagen fibers (Cf); K, keratin layer.

A histopathological examination of different treatments was analyzed. For this purpose, the epidermis, dermis, upper dermis, lower dermis, sweat glands, sebaceous glands, newly formed blood vessels, granular tissues or granulation, hair follicles, fibrous tissue or collagen fibers, and keratin layer were investigated. Our findings in the positive control showed that the keratin layer was absent, the epidermal closure was not firm, and dermal differentiation between the upper and lower layers was not evident. There were no glandular structures in the epidermis, only a few blood vessels and collagen fibers could be seen in the lower epidermis, which indicated a much slower repair process. In the “MO” group, the glandular structures of the skin were recovered but comparatively had fewer collagen fibers and angiogenesis than those of the group “MO + NT.” Moreover, keratinocytes and epidermal thickness were not fully restored. In the “NT” group, an incomplete keratin layer with ongoing re-epithelization was noticed. The glandular portion of the epidermis was not fully developed, but sweat and sebaceous were evident. Finally, we checked the “MO” and “NT” groups. Here, we found that all the structures were fully recovered with more collagen, angiogenesis, and keratin layer. The thickness of the epidermis was equal to the negative control group while tissue granulation was also optimum, indicating excellent treatment results (Figure 5).

#### The Thickness of Epidermis

**Figure 6:**
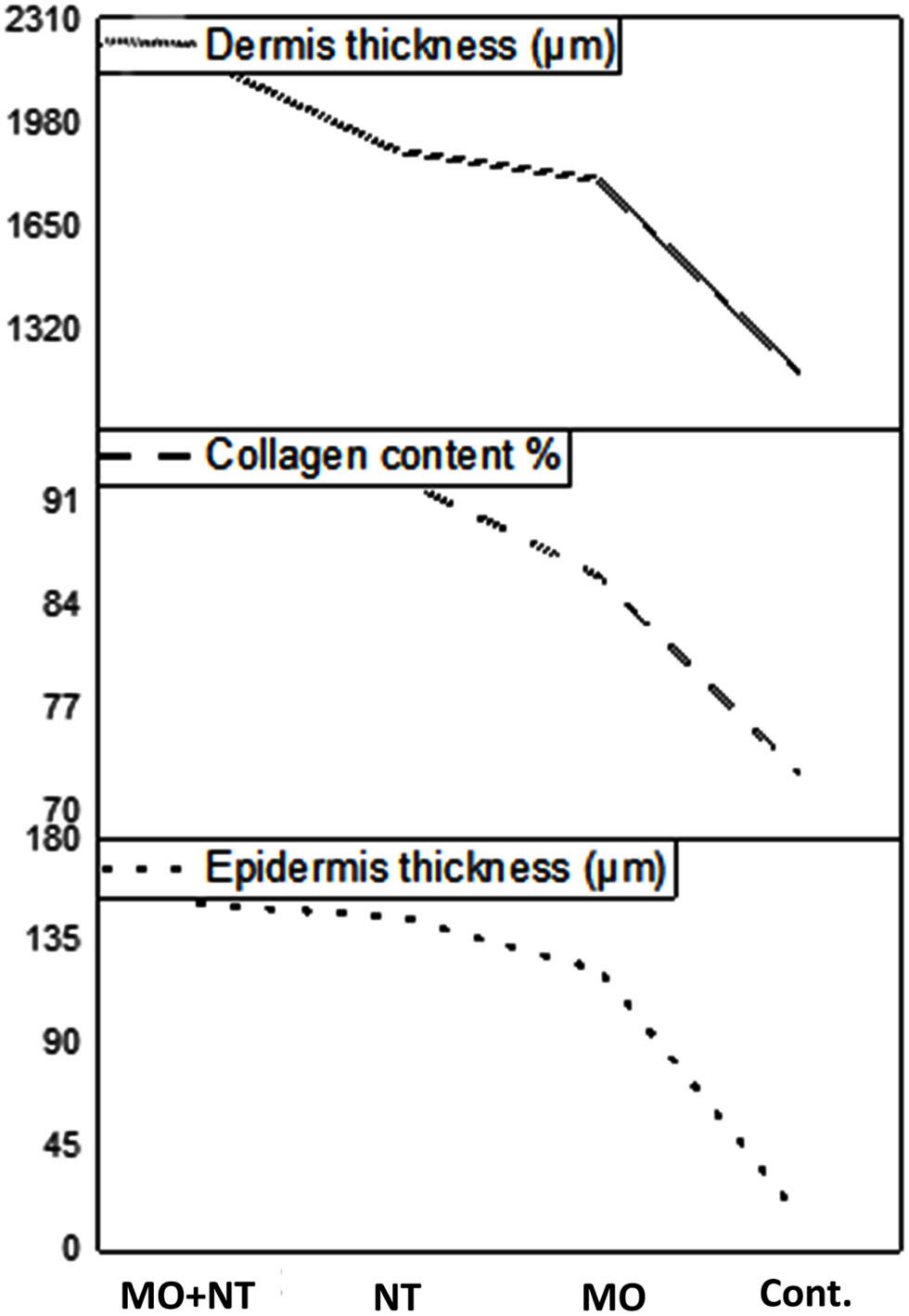
Dermis, epidermis, and collagen analyses of different treatments.

Wounds treated with “MO + NT” were found to have a higher epidermis thickness than other treatments. Concerning the epidermal thickness, all three treatment results were analyzed. It was concluded that the wounds treated with “MO + NT” had a better epidermal thickness of 151.92 μm. The result was much better in contrast to those who were treated with Manuka honey and Nitrofurazone alone. Therefore, the combination was proved to be more significant (*p* < 0.05). The honey ointment results were also better than control in epidermal thickness (Figure 6).

#### Collagen Content Percentage

Tissue samples were analyzed in the laboratory on the 28th day. “MO + NT” wounds were found to have higher collagen (*p* < 0.05) content as compared to the other wounds treated with Manuka honey and Nitrofurazone ointments. While the wounds with no treatment had loose arrangements of collagen and minimal vascularization.

#### The Thickness of Dermis

After careful examination, it was noticed that “MO + NT” has a better dermis thickness than other treatments. Statistically, G_3_ showed a significant (*p* < 0.05) improvement in the dermis as compared to the other three treatments.

## Discussion

**Figure 7:**
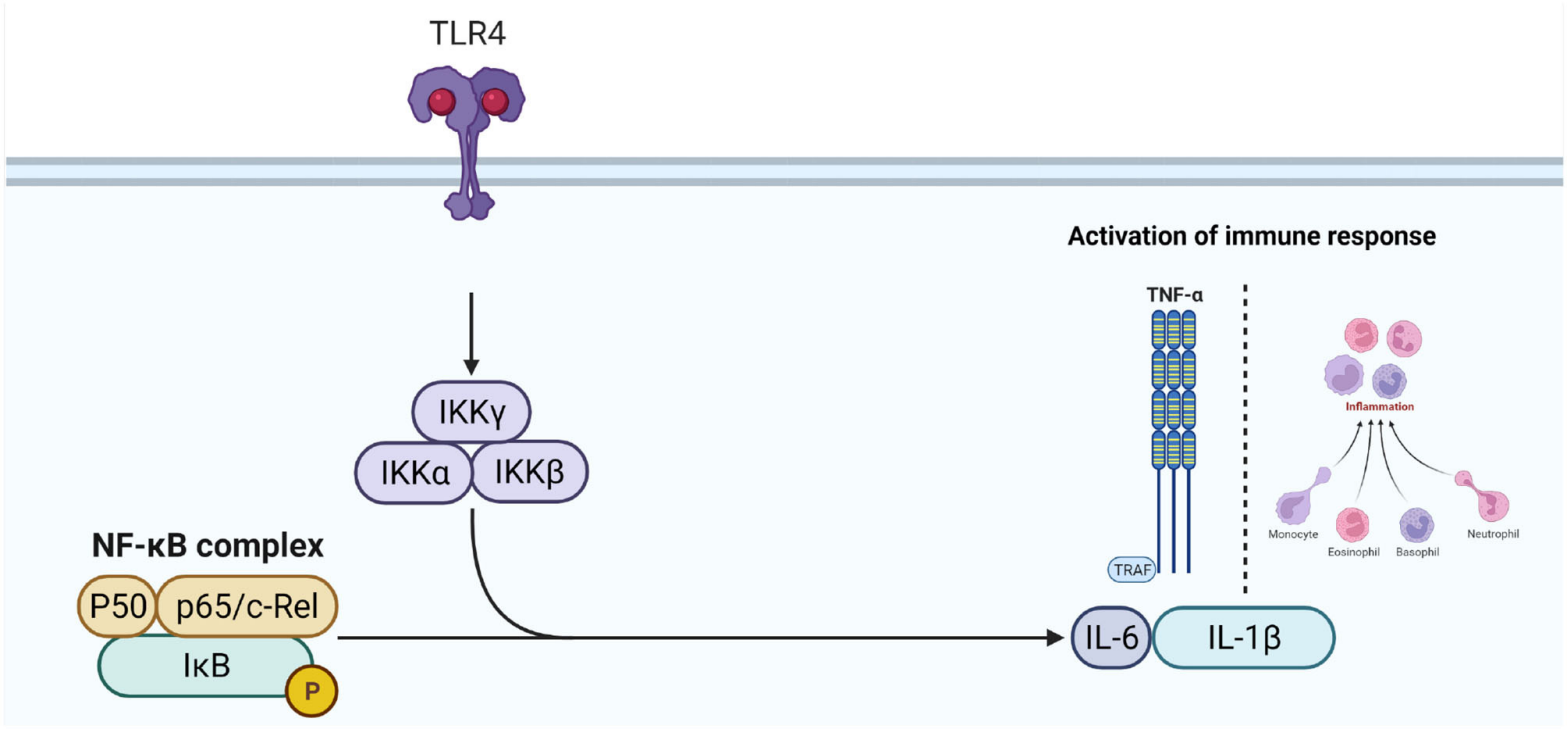
Mechanism insight of Manuka honey in the activation of inflammatory response.

The skin is the largest organ in the body that performs many important functions, e.g., homeostasis, body temperature, immunity, nervous sensations, and metabolism. The skin also acts as a physical barrier against infections (29). If this barrier is damaged, pathogens can penetrate directly into the body. Several studies have shown that burns are the leading cause of death with severe burns. Therefore, many researchers have sought to develop appropriate treatments to reduce the risk of wound infection and reduce the duration of treatment for burn injuries. These treatments include topical antimicrobials that effectively reduce mortality (30, 31), with low toxicity and sensitivity (32, 33). However, the occurrence of reactions related to allergies, toxicity, and irritation reduces skin regeneration ability, which can minimize their efficacy (29, 34). For example, the Nitrofurazone is one of the commonly used antimicrobial agents against gram-positive and gram-negative bacteria. As a consequence of its action, Nitrofurazone breaks down ribosomal proteins and some other macromolecules, which slow down the production of proteins, DNA, and RNA and stop the cellular proliferation. Additionally, it may impair bacterial cells' aerobic metabolism and carbohydrate metabolism activities (35). Meanwhile, oxidative processes produce reactive superoxide and hydroxyl radicals (36). Despite the fact that these actions take place in milliseconds or less, they have the potential to be harmful if left uncontrolled (34). As a result, the second choice is to employ natural products, which have been used since ancient times. These products are less toxic and cheaper than synthetic drugs. It has been shown that many plants and plant products effectively heal wounds. Nowadays, most drugs are a mixture of several plants or herbs, but these traditional ointments are not scientific. Using medicinal herbs has been noticed in the therapy of wounds from the very beginning due to its reduced financial load and its medical effects. Our study results are in line with the results of Bischofberger et al. (37). Where Manuka honey provided a protective barrier over the wound in preventing microbial infection. Its immunological activity accelerates wound repair and anti-inflammatory effects (38). Some other factors and bioactive components have also been involved that trigger the direct and indirect actions against the bacteria in wound repair (14). A 5.8 kDa bioactive component of Manuka honey has been identified as responsible for stimulating monocyte activity by activating the toll-like receptor 4 (TLR4). Activation of TLR4 and subsequently the intracellular signaling pathway nuclear factor kappaB (NF-κB) enhance the production of interleukin-1β (IL-1β), interleukin-6 (IL-6), and tumor necrosis factor-α (TNF-α), which are important in regulating the inflammatory response (39) (Figure 7).

Besides being able to reduce Cyclooxygenases 1 (COX-2) expression, Manuka honey can also have an antioxidant effect by changing the production of ROS and phenolic compounds (17). This is because of the production of ROS and other compounds.

Methylglyoxal is another active antibacterial ingredient of Manuka honey, which has been reported to react with lysine, arginine, and cysteine residues of structural proteins, such as collagen. Further it gives genesis to advanced glycation end products (AGEs). It promotes fibrosis in chronic tissue infections, impairs immune response microcirculation, promotes atherosclerosis and neovascularization, induces endothelial cell dysfunction, and impairs wound closure (40). Our study results are in line with the results of Bischofberger et al. (37), in which Manuka honey was found effective in experimental equine wounds in minimizing the wound area by decreasing retraction and overall healing time. The mechanism p38 is evolved during wound repair through Manuka honey. This mechanism mediates the stimulation of locomotion and proliferation by plate derivative growth factor (PDGF), the main growth factor in the early phases of wound healing (41). Our study is also in line with study of Moore et al. (42), who reviewed previous randomized controlled trials comparing honey with different treatments. Moore et al. concluded that honey is a useful treatment for burns.

The results of this study give a free hand to the companies to start the formation of medicines in the form of ointments or creams. We hope that a new burn ointment or cream can be used with herbal medicines, which will help to decrease the healing period and hypertrophic scar rate.

## Conclusion

Manuka honey ointment may provide an alternative medication for managing extensive burns. However, there are some regulatory barriers to introducing a new product containing just honey. Therefore, changing attitudes toward clinical practice might take longer due to these barriers. Such prominent factors may decrease the acceptability of such treatment option. Hence, to address such acceptability concerns, the therapeutic options must be in combination with allopathic medications. It would place more emphasis on promoting the acceptability and would be helpful in providing a better option throughout the globe. Moreover, further research with randomized prospective is also needed to fully elucidate MO's effect on burns.

## Data Availability Statement

The original contributions presented in the study are included in the article/supplementary material, further inquiries can be directed to the corresponding author/s.

## Ethics Statement

The animal study was reviewed and approved by Departmental Ethical Committee.

## Author Contributions

MK: methodology and writing original draft. KA and AA: supervision. MS, MN, and IS: reagents, materials, and analysis tools. ZB, SS, HH, and MK: writing review and editing. AC, MA, and RH: visualization. KD, YW, and KL: conceptualization, funding, and resources. All authors listed have made a substantial, direct, and intellectual contribution to the work and approved it for publication.

## Funding

This study was supported by the Start-up fund of Nanjing Agricultural University (804131) and the Start-up Fund for Distinguished Scholars of Nanjing Agricultural University (80900219).

## Conflict of Interest

KD is employed by China Tobacco Henan Industrial Co., Ltd., China. The remaining authors declare that the research was conducted without any commercial or financial relationships that could be construed as a potential conflict of interest.

## Publisher's Note

All claims expressed in this article are solely those of the authors and do not necessarily represent those of their affiliated organizations, or those of the publisher, the editors and the reviewers. Any product that may be evaluated in this article, or claim that may be made by its manufacturer, is not guaranteed or endorsed by the publisher.

## Abbreviations

PC: positive control
NS: normal saline
MO: Manuka
NT: Nitrofurazone
MO + NT: Manuka + Nitrofurazone.
